# Supplementary material for: DMRforPairs: identifying Differentially Methylated Regions between unique samples using array based methylation profiles
Source: BMC Bioinformatics. 2014 May 15;15:141. doi: 10.1186/1471-2105-15-141 (PMC4046028; doi:10.1186/1471-2105-15-141)
Supplement: Additional file 2 — DMRforPairs output for the comparison of A431-MCF7 and NA17018-NA17105. Please start from the HTML files in each folder. Available via the BMC Bioinformatics website. [file 1471-2105-15-141-S2.zip › 1394847754114233_MOESM2_ESM/A431_MCF7/figures/21810.pdf]

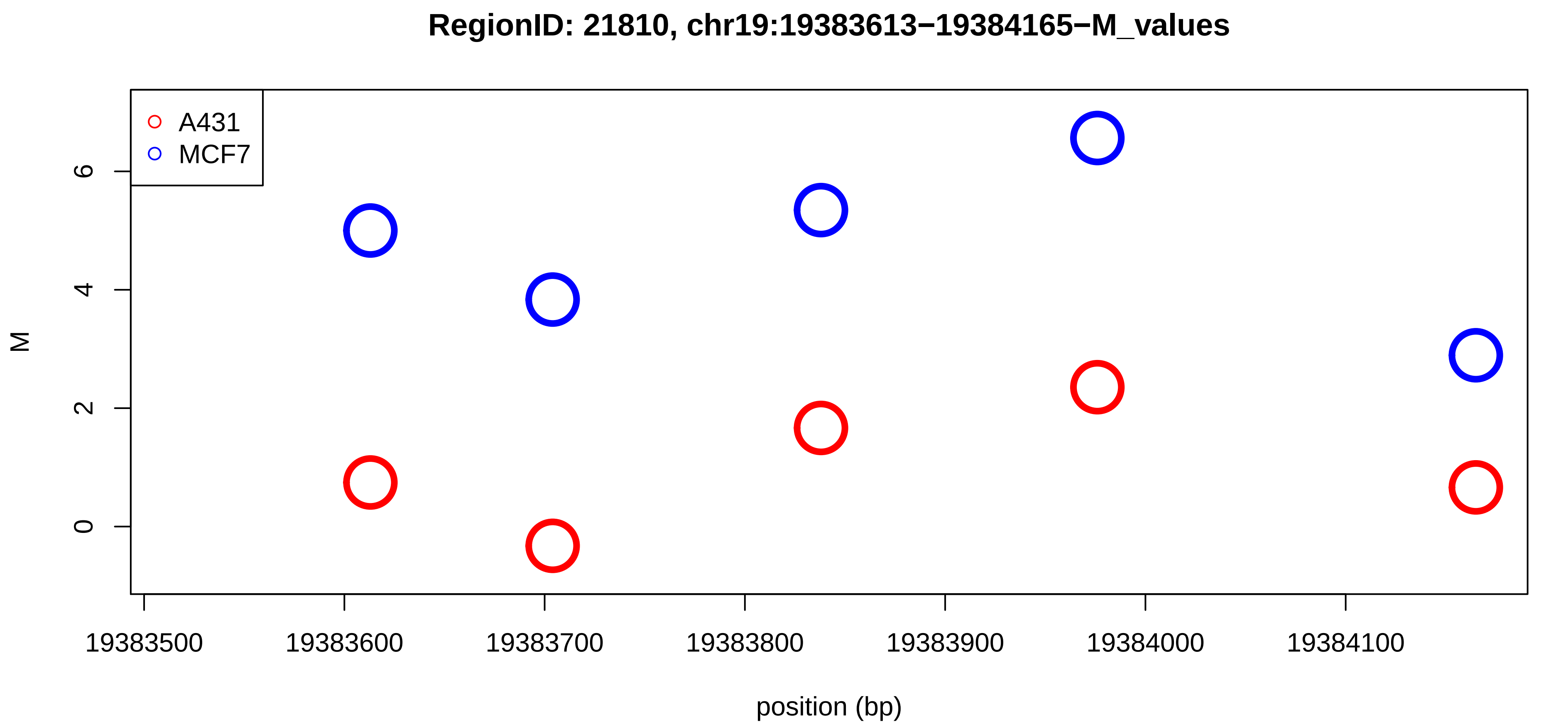

RegionID: 21810, chr19:19383613−19384165−M_values
A431
MCF7
M
0
2
4
6
19383500
19383600
19383700
19383800
19383900
19384000
19384100
position (bp)

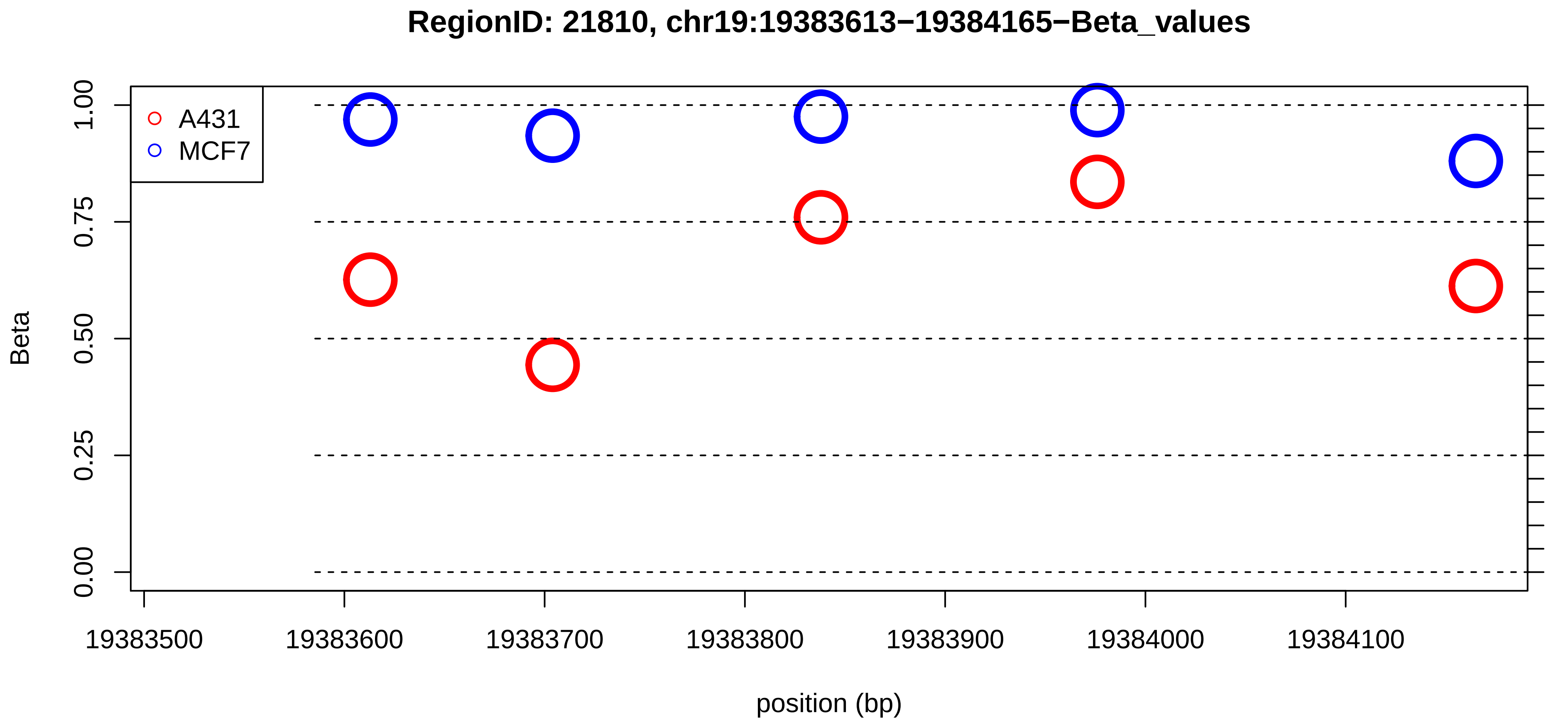

RegionID: 21810, chr19:19383613−19384165−Beta_values
A431
MCF7
Beta
0.00
0.25
0.50
0.75
1.00
19383500
19383600
19383700
19383800
19383900
19384000
19384100
position (bp)

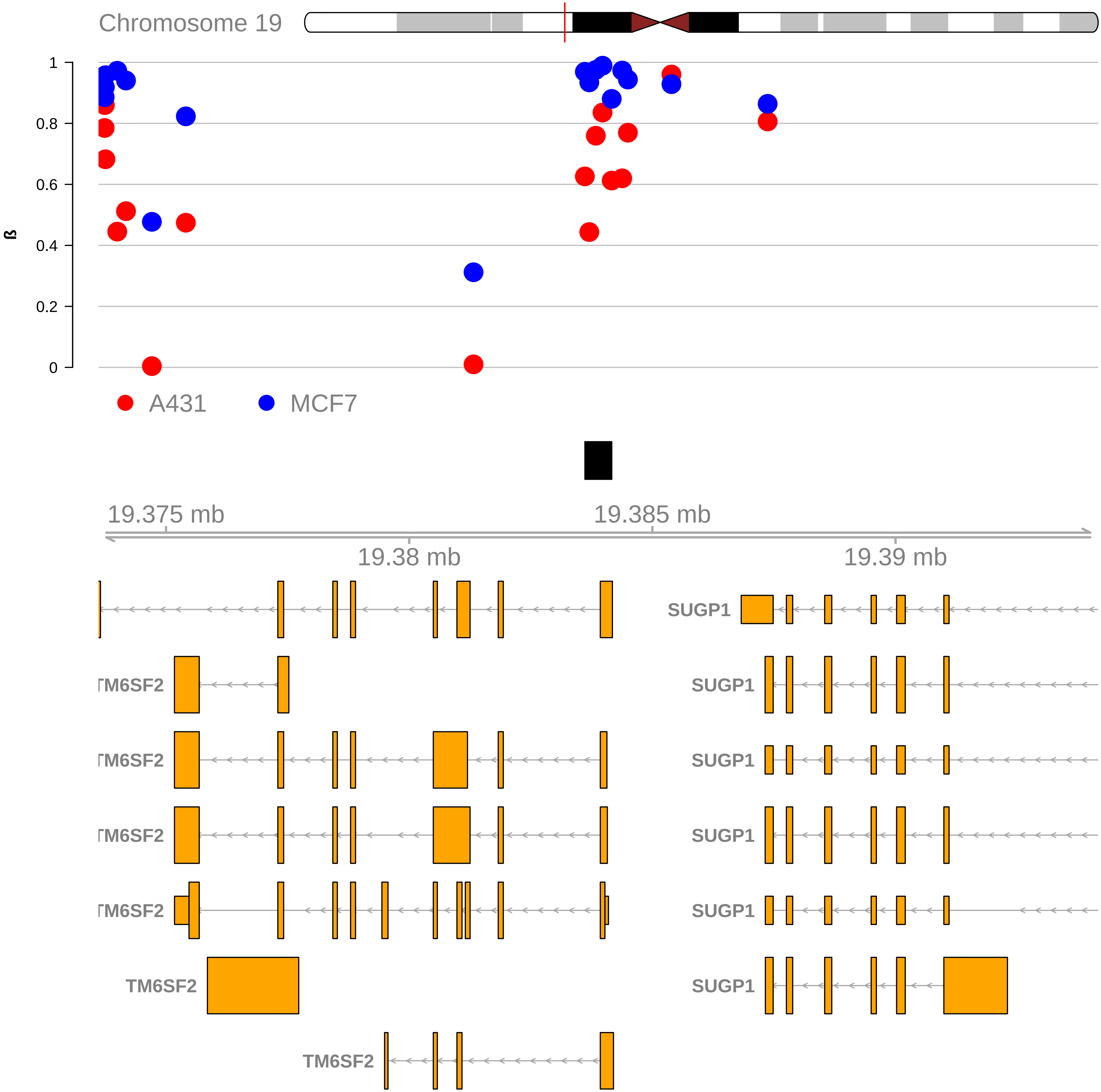

Chromosome 19
ß
1
0.8
0.6
0.4
0.2
0
A431
MCF7
19.375 mb
19.385 mb
19.38 mb
19.39 mb
TM6SF2
TM6SF2
TM6SF2
TM6SF2
TM6SF2
TM6SF2
SUGP1
SUGP1
SUGP1
SUGP1
SUGP1
SUGP1
